# Effects of Different Light Spectra on Growth Performance, Body Coloration, and Pigmentation-Related Gene Expression in Koi Carp (*Cyprinus carpio* var. *koi*)

**DOI:** 10.3390/ani16193022

**Published:** 2026-09-25

**Authors:** Xiulan Shi, Wenbin Zhu, Jianjun Fu, Zaijie Dong, Mingkun Luo

**Affiliations:** 1 Wuxi Fisheries College, Nanjing Agricultural University, Wuxi 214081, China; 2021213001@stu.njau.edu.cn; 2 Key Laboratory of Freshwater Fisheries and Germplasm Resources Utilization, Freshwater Fisheries Research Center of Chinese Academy of Fishery Sciences, Ministry of Agriculture and Rural Affairs, Wuxi 214081, China; zhuwb@ffrc.cn (W.Z.); fujj@ffrc.cn (J.F.)

**Keywords:** all-red *koi*, different light spectra, growth, body coloration

## Abstract

Light is a critical environmental factor in aquaculture, directly or indirectly regulating the growth, reproduction, body coloration, and biological rhythms of cultured animals. However, in *koi* farming, spectral composition is often overlooked or arbitrarily selected. This study aimed to evaluate the effects of different LED spectra (red light “RL”, blue light “BL”, yellow light “YL”, white light “WL”) and natural light (NL) on the growth performance, body coloration, and underlying molecular regulatory mechanisms of all-red *koi* (*Cyprinus carpio* var. *koi*). The results showed that WL treatment significantly suppressed the weight gain rate and specific growth rate of *koi*. After 60 days of BL exposure, the condition factor significantly increased, whereas YL, WL, and RL led to varying degrees of reduction. In terms of body color, BL significantly increased skin lightness (L* value) and promoted the deposition of lutein and *β*-carotene in the skin, while upregulating the expression of pigmentation-related genes such as *gch* (GTP cyclohydrolase), *scarb* (Scavenger receptor class B), *oca*2 (Oculocutaneous albinism II), and *xdh* (Xanthine dehydrogenase). Meanwhile, the calcium signaling, MAPK signaling, and TGF-*β* signaling pathways were activated, synergistically mediating light adaptation, metabolic regulation, and pigmentation. However, chronic or excessive BL irradiation exacerbated dermal loosening and vacuolation, decreased a* and b* values, and was thus unfavorable for color maintenance. In contrast, long-term exposure to RL and WL tended to inhibit growth and cause dermal tissue loosening, whereas YL was relatively beneficial to fish health but had limited color-enhancing effects. Overall, BL is the optimal spectrum for short-term (30–60 days) color enhancement, and long-term exposure to RL and WL should be avoided. In practical production, a cyclic lighting regimen is recommended, i.e., short-term BL for color enhancement followed by recovery under NL or YL. This study provides an important theoretical basis for optimizing the light environment for *koi* culture, improving the quality of ornamental *koi*, and enhancing aquaculture production efficiency.

## Simple Summary

Through exploring different LED spectral environments and natural light cultivation modes, we obtained optimized light regimens for the growth and coloration of all-red *koi*, addressing the issues of arbitrary light selection and inefficient light-environment utilization in *koi* farming. To elucidate the effects and underlying mechanisms of light spectra on growth and pigmentation, we comprehensively analyzed growth performance, skin color characteristics, and gene expression patterns. Our findings confirmed that blue light effectively promotes short-term color enhancement; however, long-term exposure to a single spectrum adversely affects growth and skin integrity.

## 1. Introduction

In recent years, light management has garnered widespread attention and application in fields such as agriculture and environmental protection. Artificial illumination is extensively employed in greenhouse cultivation, aquaculture, and smart agriculture to enhance efficiency and quality. A suitable aquaculture environment is crucial for achieving efficient, healthy, and sustainable development in aquaculture, as well as for improving the welfare of farmed fish. As a critical environmental factor in aquaculture, light directly or indirectly influences animal growth and development, reproduction, body coloration, behavior, and biological rhythms [1].

Most fish possess the ability to perceive their environment and distinguish colors, exhibiting preferences or aversions to different light spectra. For instance, silver carp (*Hypophthalmichthys molitrix*) show a significant preference for full-spectrum and blue light environments compared to red and green light [2], while juvenile rainbow trout (*Oncorhynchus mykiss*) prefer blue light conditions [3]. Phototaxis preference for specific colors helps fish respond rapidly to external environments, reduce the negative impacts of predation risk and stress, and allocate more energy to growth and reproduction [4,5,6]. For instance, Nile tilapia demonstrates improved growth characteristics and disease resistance when exposed to appropriate light intensities [7]. Photoperiod manipulation has been found to regulate gonadal development in Atlantic salmon (*Salmo salar*) [8], while specific light conditions have been shown to enhance the specific growth rates of Malabar grouper (*Epinephelus malabaricus*) [9]. Additionally, Haddock (*Melanogrammus aeglefinus*) smolts exhibit higher survival rates in blue and green light environments [10].

Recognizing the importance of sustainable aquaculture practices, the Food and Agriculture Organization of the United Nations (FAO) has introduced the concept of ‘green aquaculture’. This approach encourages the adoption of low-carbon, high-efficiency, and environmentally friendly technologies. Light-emitting diodes (LEDs), with their controllability, corrosion resistance, low pollution, and diverse color characteristics, offers unprecedented technological possibilities for optimizing the light environment in ornamental fish farming, garnering widespread attention and application [11]. Shin et al. reported that short-wavelength green and blue lights enhanced antioxidant activity in the yellowtail clownfish (*Amphiprion clarkia*), while red light impaired its physiological functions and induced oxidative stress [12]. Heydarnejad et al. (2017) found that yellow light improved growth performance and reduced the cortisol response induced by stress in pearl fish (*Trichopodus leerii*) [13].

*Koi* carp (*Cyprinus carpio* var. *koi*) are highly valued for their stunning body colors and unique patterns, which have both ornamental and economic significance [14,15]. Previous studies have demonstrated that light spectrum serves as a critical environmental signal regulating growth and non-specific immunity in common carp. Appropriate spectral conditions can improve growth performance, whereas unsuitable illumination may trigger stress responses and suppress immune competence [16]. Bairwa et al. reported that blue and green light elevated lysozyme activity and respiratory burst activity in *koi* carp, while yellow–red light could induce immune suppression under certain aquaculture conditions [17]. The skin pigments of *koi* carp constitute a complex mixture, mainly consisting of carotenoids (lutein, *β*-carotene, zeaxanthin), pteridine-derived pigments, and melanin. The characteristic reddish-orange skin phenotype of all-red koi primarily originates from the tissue deposition of dietary-derived carotenoids, because teleost fish are incapable of de novo carotenoid biosynthesis [18]. Carotenoids generally exert photoprotective effects on cell membranes to which they bind: they capture excess radiation and dissipate energy in the form of heat, thereby preventing peroxidative damage to biomolecules including DNA, sugars, proteins, and lipids. Furthermore, these pigments may help maintain epidermal-barrier integrity and participate in mucosal immune homeostasis [19,20].

The all-red *koi* is a variant that has undergone long-term artificial selection and breeding improvement. In traditional Chinese culture, all-red *koi* is regarded as a symbol of good fortune, representing wealth, happiness and longevity. Given its high stability in body color variation, all-red *koi* serves as an excellent model for studying pigment cell development and pigment synthesis. Furthermore, it plays a crucial role in aquaculture production and genetic breeding [18,21]. Previous studies regarding light in *koi* carp have provided some foundational insights, most have primarily focused on the effects of photoperiod and light intensity on growth and immune responses. Significant research gaps persist concerning the regulation of body coloration and the underlying molecular mechanisms. Therefore, this study conducted a long-term rearing experiment with four representative monochromatic LED lights (blue, white, yellow and red) and natural light as the control. This study aims to provide new insights into the environmental control of pigmentation in *koi* carp, revealing additional details of environmental responsiveness within the molecular regulatory mechanisms governing growth and body color, thereby providing a scientific foundation for improving production efficiency of *koi*.

## 2. Materials and Methods

### 2.1. All-Red Koi Rearing System and Light Management

The all-red *koi* (N = 225, average weight = 8.62 ± 0.02 g) were obtained from the aquaculture base of Jiangsu Qihong Ecological Agriculture Development Co., Ltd. (Taicang, China) and transported to the aquaculture facility of the Freshwater Fisheries Research Center, Chinese Academy of Fishery Sciences in a 256 L recirculating aquaculture system at 24 ± 1 °C. The fish were stocked in a recirculating aquaculture system (RAS, 256 L) at 24 °C and individually tagged with Passive Integrated Transponders (PIT). Five light treatments were established: white LED (WL, full spectrum, 400–770 nm), yellow LED (YL, 570–590 nm), blue LED (BL, 450–470 nm), red LED (RL, 620–750 nm) (Cat. No. JY-B4832, Jiuyan, Beijing, China), and a natural control group. Light sources were randomly assigned and fixed 30 cm above each tank. Three replicate tanks were set for each light treatment, with 15 fish stocked per tank (15 tanks in total). The total irradiance for all light treatments was set to (240 ± 20) mW m^−2^. Each rearing tank was covered with an independent black shading cover to eliminate ambient light interference and ensure the stability of experimental light conditions. Irradiance detection and calibration were performed every two days using a spectroradiometer (SRI-2000UV, Shangze, Taiwan, China). The photoperiod was set at 12 h light and 12 h dark (12L:12D). Fish were fed three times daily (at 6:00, 12:00, and 18:00) with a commercial diet for the 3-month experimental period at a feeding rate of 5% of total body weight per day, adjusted biweekly based on fish weight. The recirculating system was equipped with oxygenation and filtration devices.

### 2.2. Determination of Carotenoids and Lutein

*β*-carotene was measured in this study. The samples were pre-processed according to the previous method [22] before loading onto the machine. The concentrations of the components were evaluated using chromatography (DGLC, Thermo Fisher Scientific, Waltham, MA, USA) at 445 nm. The chromatographic column used was a YMC carotenoid S-3 μm (250 × 4.6 mm) liquid chromatography column, and the mobile phase consisted of mobile A (MeOH) and B (MeOH: MTBE: H_2_O = 20:75:5). The flow rate was at 1.0 mL/min, and the elution gradient followed this pattern: 0 min A: B (85:15 *v*/*v*), 5 min A:B (75:25 *v*/*v*), 10 min A:B (70:30 *v*/*v*), 22 min A:B (55:45 *v*/*v*), 24 min A:B (85:15 *v*/*v*), 26 min A:B (85:15 *v*/*v*). HPLC analysis for lutein was conducted according to previous studies [23], elution was performed with 15% methanol and 85% acetonitrile for 20 min. Flow rate was 0.7 mL/min, detection wavelength was 450 nm, injection volume was 20 μL, and the temperature was 30 °C.

### 2.3. Indicator Measurement and Sample Collection

During the culture experiment, samples were collected on the 30th, 60th, and 90th day of rearing, respectively. All experimental fish were fasted for 24 h before sampling. The experimental fish were anesthetized with MS-222 (60 mg/L, Sigma, St. Louis, MO, USA) to alleviate stress. Individual growth performance, including weight gain rate (WGR), specific growth rate (SGR), and condition factor (CF), were recorded for each PIT-tagged fish. Fish were weighed and the final body weight (FBW) was calculated. Growth performance and feed utilization were calculated according to the following formulae: Weight gain (WG, g) = FBW − IBW, Weight gain rate (WGR, %) = 100 × [(FBW − IBW)/IBW], Specific growth rate (SGR, %/day) = 100 × [(lnFBW − lnBW)/t] and Condition factor (K, g/cm^3^) = 100 × FBW/L^3^. Where FBW and IBW are the final and initial body weights (g), respectively; t is the experimental duration (days); and L is the final total body length (cm).

Skin color parameters (L*, a*, b*) were measured on the dorsal, ventral and fin regions with a colorimeter (NH300, Shenzhen, China). L* stands for lightness (high values = bright/white; low values = dark/black). Positive a* values indicate redness (negative for green), and positive b* values indicate yellowness (negative for blue). Whiteboard calibration was carried out prior to measurement. The instrument was tightly pressed against the sampling site to record readings. To ensure the accuracy of the measurements, the fish were placed on a uniform background to minimize the influence of external light according to the previous method of Wang et al. [24]. Six fish were randomly selected from each group for skin tissue collection (dorsal and ventral regions). These samples were rinsed with sterile water, flash-frozen in liquid nitrogen, and stored at −80 °C for real-time quantitative PCR (RT-qPCR) and transcriptomic sequencing. Additionally, skin tissues from the dorsal and ventral regions of three fish were collected, flattened to maintain morphological integrity, fixed in 4% paraformaldehyde, and stored at room temperature.

### 2.4. Histological Staining

Skin tissues were fixed in 4% paraformaldehyde for 24 h, dehydrated through a graded ethanol series, cleared in xylene, and embedded in paraffin. The paraffin-embedded tissues were sectioned longitudinally, and the sections were stained with hematoxylin and eosin (HE), then mounted with neutral resin. Hematoxylin stained the nuclei blue-purple, while eosin stained the cytoplasm red. The stained sections were observed and imaged using an Olympus BX51 microscope (Kyoto, Japan) with AxioCam camera (Zeiss, Jena, Germany). The detailed procedures were the same as what was outlined in the publication by Luo et al. [25].

### 2.5. Transcriptome Data Analysis

TRIZOL (Cat. No. 15596026, Invitrogen, Carlsbad, CA, USA) was used to extract the sample’s total RNA, and DNase I (Cat. No. EN0521, ThermoFisher Scientific Inc., Waltham, MA, USA) was used to remove genomic DNA. RNA quality was assessed by 1% agarose gel electrophoresis and an Agilent Bioanalyzer 2100 system (Model G2939BA, Agilent Technologies, Santa Clara, CA, USA). The total RNA concentration of each sample was measured with a NanoDrop 2000c spectrophotometer (Model NanoDrop^®^ ND-1000, ThermoFisher Scientific Inc., Waltham, MA, USA). Samples were prepared using the NEB Next^®^ UltraTM RNA Library Preparation Kit for Illumina (Cat: No. E7770, New England Biolabs, Ipswich, MA, USA). The libraries were then sequenced on Illumina’s Nova seq 6000 platform, and 150 bp paired-end reads were generated following the method of Liang et al. [26].

Raw data were quality assured using Fastp (v0.20.0) (https://github.com/OpenGene/fastp accessed on 20 September 2024). High quality reads were aligned to the C. carpio reference genome (Accession number: ASM1834038v1) using HISAT 2 (v2.1.0) (http://daehwankimlab.github.io/hisat2, accessed on 21 August 2026) to generate read count files of transcripts and annotated with known NCBI transcripts [27]. Differential expression analysis was performed using the DESeq2 R package (http://www.bioconductor.org/packages/release/bioc/html/DESeq2.html accessed on 15 October 2024) (version 1.44.0) based on raw read counts, utilizing its built-in normalization algorithm. FPKM (Fragments Per Kilobase of transcript per Million fragments mapped) values were calculated exclusively for gene expression visualization and descriptive statistics. Genes with |log_2_ (fold change)| ≥ 2 and an adjusted *p* < 5 × 10^−6^ were identified as differentially expressed genes (DEGs). Functional annotations were performed using the Swiss-Prot, GO, KEGG, Clusters of Orthologous Groups (COG), Eukaryotic Ortholog Groups (KOG), Pfam, and Non-Redundant (NR) databases [28]. DEGs were analyzed via the STRING database (https://string-db.org/ accessed on 25 October 2024) to obtain protein–protein interaction (PPI) networks. These networks were subsequently visualized and refined using the STRING plugin within the Cytoscape software (v3.6.1) platform.

### 2.6. Validation of RNA-Seq and Further Investigation of Candidate Genes

A total of 18 DEGs from the significantly enriched KEGG pathway were analyzed to confirm the RNA-seq results by qPCR. The steps for qRT-PCR refer to our previously study [29]. RNA was extracted from the samples using TRIZOL (Cat. No. 15596018, CWBIO, Beijing, China). The quantitative real-time PCR (qPCR) and reverse transcription procedures followed the same sequential steps as described in our previous study [19]. Relative expression values were normalized against *β*-actin using the 2^−△△Ct^ method. All primers (Table S1) were designed using Primer Premier v5.0 (Premier Biosoft, Palo Alto, CA, USA) and synthesized by Sangon Biotech, Shanghai, China. Values were presented as mean ± SD of three independent biological replicates (each biological replicate represented skin tissue from distinct tanks and measured in technical replicates; the mean value of technical replicates was used for subsequent statistical analysis). All data were calculated with SPSS 22.0 (SPSS Inc., Chicago, IL, USA) using one-way analysis of variance (ANOVA) followed by Duncan tests (significance threshold with *p* < 0.05).

### 2.7. Statistical Analysis

Statistical analysis was performed using SPSS v19.0, and all data are expressed as mean ± SD. All individual fish measurements were averaged to generate tank-level values prior to statistical testing, with the tank defined as the experimental unit to avoid pseudoreplication. For data collected at each sampling time point (30, 60 and 90 days), one-way ANOVA was applied to evaluate the effects of light spectrum, followed by Duncan’s multiple range test for post hoc comparisons among the five light treatments. A two-tailed unpaired Student’s *t*-test was used only for pairwise comparisons between two groups. For time-series data obtained from the same PIT-tagged individuals across the three sampling time points, repeated-measures ANOVA was adopted. In this model, light treatment was set as the between-subject factor and sampling time as the within-subject factor to account for correlations among repeated measurements from identical individuals. Differences were considered statistically significant when *p* < 0.05.

## 3. Results

### 3.1. Effect of Illumination on Growth Performance and Coloration of All-Red Koi

The WGR and SGR value of all-red *koi* reared under white light were significantly lower than those of NL group (*p* < 0.05), while no significant changes were observed in the two parameters for groups treated with other light colors after 30 days of culture. After 60 days of culture, the CF value of individuals in YL, WL and RL group was significantly lower than that of NL group (*p* < 0.05), while the CF value of the BL group was significantly higher than that of all other treatment groups (*p* < 0.05) (Figure 1A).

Lab colorimetric measurements revealed significant differences in the chromaticity of all-red *koi* skin among the groups after 90 days of culture. Compared to NL group, L* values of the dorsal skin in other groups were significantly increased (*p* < 0.05). After 30 days of culture, a* values of the dorsal in YL, BL, WL and RL groups were significantly lower than those of NL group (*p* < 0.05). b* values of the dorsal of YL group were significantly lower than those of other groups (*p* < 0.05). After 90 days of culture, a* values of the dorsal in the BL group were also significantly reduced compared to other groups (*p* < 0.05). Throughout the entire culture period, b* values of the dorsal in WL group remained at the highest level, significantly exceeding other groups (*p* < 0.05) (Figure 1B).

Regarding the ventral skin, after 30 days of culture, the L* values of NL group were significantly lower than those of other groups (*p* < 0.05), with BL group showing significantly higher than the RL, YL and WL groups (*p* < 0.05). The a* values in NL group were significantly higher than those of other groups (*p* < 0.05). The b* values in NL group were significantly higher than those of other groups (*p* < 0.05). After 90 days of culture, L* values in NL group were also significantly lower than those of other groups (*p* < 0.05). The a* and b* values in BL group were significantly lower than those of other groups (*p* < 0.05) (Figure S1A). In terms of fins skin, after 30 days of culture, the b* values in the RL group were significantly reduced compared to other groups (*p* < 0.05). After 60 and 90 days of culture, the L* values in BL group were significantly higher than those of other groups, reaching the peak (*p* < 0.05). Compared to other groups, a* values in the BL group were significantly lower (*p* < 0.05). The b* values of WL group were significantly increased compared to NL, RL and BL group (*p* < 0.05) (Figure S1B).

### 3.2. Histological Responses of Skin Structure in All-Red Koi Exposed to Different Light Spectra

The results of Hematoxylin-Eosin (HE) staining revealed that rearing duration and light spectrum jointly modulated the skin histological structure in the dorsal and ventral regions of all-red *koi*, and obvious regional differences existed in the skin response to light treatments. After 30 days of culture, intact skin structure and densely arranged collagen fibers were observed in all groups, with minor intergroup histological differences. As the culture period extended to 60 days and 90 days, gradual morphological alterations occurred in the dermis of each group. The NL group and YL group exhibited orderly, arranged collagen fibers and low vacuolation throughout the experiment, maintaining favorable tissue integrity. Dermal fibers in WL group gradually loosened with prolonged culture. Pronounced histological variations were found in RL and BL groups: enlarged connective tissue spaces, disordered fiber arrangement and dramatically increased vacuolation emerged during the middle and late culture stages, resulting in remarkable disruption of skin structure, especially in RL group. Under identical light conditions, ventral skin was thinner than dorsal skin, and spectrum-induced tissue loosening and vacuolation occurred earlier and were more severe in ventral skin (Figure 2 and Figure S2).

### 3.3. Effects of Light Spectra on Skin Carotenoid and Lutein Concentrations in All-Red Koi

Results revealed that lutein content significantly increased after 30 days under blue light, and this trend of increase continued, with levels at 60 days being significantly higher than those in all other groups (*p* < 0.05). For β-carotene, a significant increase was observed at 30 days in BL, RL, and YL group (*p* < 0.05), with levels significantly surpassing other groups. Although its content under blue light decreased at 60 days, it remained significantly higher than in other groups at this time point. By the end of the 90-day period, no significant differences were found in the content of either lutein or β-carotene among any of the light treatment groups (Figure 3).

### 3.4. Gene Expression of Skin Color-Related Genes Under Different Light Spectra

The results of qPCR showed that different light spectrum treatments significantly altered the expression levels of body color-related genes in the dorsal and ventral skin of all-red *koi* carp. Gene expression exhibited distinct temporal dynamic characteristics and tissue-specific differences (Figure 3). In the dorsal skin, the expression of *oca*2 (oculocutaneous albinism II), *gch* (GTP cyclohydrolase), *scarb* (scavenger receptor class B member), *pax*3 (paired box 3) and *xdh* (xanthine dehydrogenase) was all significantly higher at 30 days in the BL group than in the NL group at 30 days (*p* < 0.01). In addition, the expression patterns of the iridocyte marker gene *pnp*4*a* (purine nucleoside phosphorylase 4a) and *cd*36 (cluster determinant 36) were similar across all spectral ranges, showing an initial decrease followed by an increase, although levels remained lower than those in the NL group. The expression of the gene *cbp* (CREB-binding protein), which is related to carotenoid uptake and catabolism, was consistently lower than that in the NL group across all spectral ranges. Similarly, the expression of the xanthophores development gene *pax*7 (paired box 7), was only significantly upregulated at 60 days in the BL group, while maintaining low expression levels in all other light groups (Figure 4A). The magnitude of gene responses was markedly higher in ventral skin, suggesting higher sensitivity to spectral treatments. Gene expression levels in ventral skin tended to be higher than those NL group and were sustained for longer duration. The expression of *gch*, *oca*2, *cd*36, *pax*3, *pax*7, *pnp*4*a*, *cbp*, *xdh* and *scarb* peaked at 30 days in the BL, YL and WL groups (Figure 4B).

Based on the above results, we found that BL significantly improved the condition factor and promoted the accumulation of lutein and *β*-carotene, showing prominent phenotypic advantages. Therefore, we further performed transcriptomic sequencing on the BL group.

### 3.5. RNA-Seq Analysis

After quality control, a total of 64.92 Gb of clean data was obtained from the skin tissue transcriptome sequencing, with at least 6.10 Gb per sample and an average Q30 base percentage exceeding 92.26%. Sequence alignment against the reference genome revealed a mapping rate of over 85.57% for all samples (Table S2). Pearson correlation coefficients greater than 0.8 indicated good consistency among the biological replicates (Figure S3A), which was further corroborated by principal component analysis (PCA) (Figure S3B). A total of 58,961 expressed genes were identified, comprising 57,633 known and 1328 novel genes. Subsequent analysis of differentially expressed genes (DEGs) revealed 5377 upregulated and 4784 downregulated genes in the BL0_vs_BL30 group, 2437 upregulated and 1299 downregulated in BL0_vs_BL90, and 2223 upregulated and 1780 downregulated in BL30_vs_BL90 (Figure 5A). Furthermore, 5356, 1199, and 752 unique DEGs were identified in these respective comparisons, with 247 DEGs shared across all three groups (Figure 5B). To elucidate the underlying regulatory mechanisms, we identified hub genes using MCODE and CytoHubba (Figure 5C,D), including *cytb* (cytochrome b), *cox* (cytochrome c oxidase subunits), *polr*1*b* (RNA polymerase I subunit B), *trub*2 (tru-based rna 2), *rbm*34 (RNA binding motif protein 34), *nd* (NADH dehydrogenase subunits), *ddx*56 (DEAD-box helicase 56) and bop1 (Block of proliferation 1). These hub genes, which are involved in critical biological processes such as RNA processing, ribosome assembly, energy metabolism, and transcriptional regulation, were significantly upregulated in the BL30 group compared to the BL0 and BL90 groups (Figure 5E).

### 3.6. GO and KEGG Enrichment Analysis

To further understand DEGs function under the light treatment, all comparative groups DEGs were subjected to GO and KEGG enrichment analysis. Functional annotation was performed based on seven databases, including Swiss-Prot, GO, KEGG, COG, KOG, Pfam, and NR. The largest number of annotated genes was obtained from the Pfam and NR databases (Figure S4). Subsequently, GO and KEGG enrichment analyses were conducted on DEGs from all comparison groups.

For the BL30_vs_BL90 group, the top 20 enriched GO terms for upregulated and downregulated genes were displayed. In the cellular component category, the dominant terms were nucleolus (GO: 0005730), small-subunit processome (GO: 0032040) and extracellular matrix (GO: 0005578). The main molecular function terms contained RNA binding (GO: 0003723), ATP-dependent RNA helicase activity (GO: 0004004) and nucleic acid binding (GO: 0003676). The biological process terms included tRNA processing (GO: 0008036), ribosome biogenesis (GO: 0042254) and regulation of cell growth (GO: 0001558) (Figure 6A). KEGG enrichment analysis revealed significant enrichment in several pathways, such as focal adhesion, ribosome biogenesis in eukaryotes, ECM–receptor interaction, and the PPAR signaling pathway (Figure 6B). In the BL0_vs_BL90 group, significantly enriched GO biological processes covered actin filament organization (GO: 0007015), sarcomere organization (GO: 0045214) and muscle contraction (GO: 0006936). The enriched cellular component terms included mitochondrial inner membrane (GO: 0005743), muscle myosin complex (GO: 0005859) and troponin complex (GO: 0005861). KEGG pathways including spliceosome, ribosome, oxidative phosphorylation and cardiac muscle contraction were significantly enriched (Figure S5A).

In the BL0_vs_BL30 group, GO terms were significantly enriched in cellular component, biological process and molecular function. Representative terms were nucleolus (GO: 0005730), structural constituent of ribosome (GO: 0003735), spliceosomal complex (GO: 0005681), mitochondrial inner membrane (GO: 0005743), nucleotide binding (GO: 0000166) and sarcoplasmic reticulum membrane (GO: 0033017). Meanwhile, DEGs were significantly enriched in KEGG pathways of oxidative phosphorylation, calcium signaling pathway, cardiac muscle contraction and pyruvate metabolism (Figure S5B).

To further explore the functions of these DEGs, we performed GO and KEGG enrichment analyses. KEGG pathway analysis revealed significant enrichment in Environmental information processing, specifically within the subcategories of signal transduction and signaling molecules and interaction (Figure 7A). Within these pathways, we identified several genes closely associated with body coloration and growth that were significantly upregulated across the comparison groups including *cnn*1*b* (cofilin-1, b), *mitf* (microphthalmia-associated transcription factor), *bco*2*b* (beta-carotene oxygenase 2b), *pax*7 (paired box 7), *scarb* (scavenger receptor class B), *rdh*12 (retinol dehydrogenase 12), *igfbp*1 (insulin-like growth factor binding protein 1), *fgfbp*2-*like* (fibroblast growth factor binding protein 2-like), *igf*2*b* (insulin-like growth factor 2b), *kitlg* (kit ligand), *fzd*10 (frizzled class receptor 10), and *lef*1 (lymphoid enhancer-binding factor 1) (Figure 7B). Furthermore, Protein–Protein Interaction (PPI) network analysis revealed that the genes involved in Environmental information processing were primarily clustered within the calcium, MAPK, and TGF-*β* signaling pathways. These pathways showed transcriptional interconnection according to enrichment and PPI network analyses. The results indicate a potential coordination pattern: calcium signaling was associated with rapid cellular responses, while MAPK and TGF-*β* signaling were linked to long-term developmental and functional regulation. The data also suggest putative synergistic interactions among these pathways, which may participate in core biological processes including growth, development, and melanogenesis (Figure 7C).

To verify the reliability of the transcriptome sequencing, 18 DEGs were randomly selected from three comparison groups for qRT-PCR analysis. The results demonstrated that the expression patterns of these 18 genes were consistent with the RNA sequencing results, indicating the reliability and accuracy of the transcriptome data (Figure S6). Notably, many skin-pigmentation genes (e.g., *pax*7, *scarb*, etc.) from our preliminary qPCR screening also exhibited consistent expression trends within this dataset.

## 4. Discussion

Light is one of the crucial environmental factors influencing fish growth and development which rely on natural light to regulate their circadian rhythms. Nowadays, aquaculture environments are often contaminated by artificial light sources, particularly in the ornamental fish market. To enhance the visual appeal of ornamental fish during cultivation and sale, artificial light sources are frequently chosen arbitrarily. However, artificial light could interfere with physiological processes such as feeding, reproduction, and growth [30,31]. Given the differences in fish visual characteristics and their sensitivity to light conditions, various light spectra have distinct impacts on fish growth and development. Nevertheless, the molecular mechanisms underlying different wavelengths of artificial light induce abnormal pigmentation and growth inhibition remain poorly understood. In the present study, no significant differences in WGR and SGR were observed among red, yellow and blue light treatments after 90-day culture compared with the natural-light control. By contrast, both WGR and SGR decreased significantly under white-light exposure at 30 d, suggesting that certain spectra exert growth-inhibitory effects on all-red *koi* under short-term light treatment. Pir et al. reported adverse developmental impacts of white and red light on zebrafish, whereas blue light exhibited the least negative influence [32]. Our results also support that long-term spectral treatments produce negligible effects on the growth performance of all-red *koi* [33,34].

All-red *koi* are popular ornamental fish for their brilliant body color and elegant morphology. Condition factor and body proportion directly determine their market value and serve as key evaluation indices in aquaculture. Significant differences in condition factor were detected across spectral groups at 60 days of culture, and blue light markedly increased the condition factor of all-red *koi*. This may be partly explained by the superior water-penetrating capacity of blue light, while long-wave spectra such as red light attenuate rapidly in water. In addition, previous studies have confirmed that blue light can effectively stimulate the feeding behavior of fish [35]. Bayarri et al. also pointed out that sea bass cultured under short-wavelength blue light have reduced melatonin secretion and maintain longer awake time, which eventually leads to increased feeding rate [36]. The present study demonstrated that the skin lightness of all-red *koi* increased under all spectral treatments over the 90-day culture period. The effects of light environment on fish body color are mediated by neuroendocrine status, reflecting adaptive modifications of fish to varied light conditions. Previous studies have demonstrated that prolonged photoperiod increases skin lightness and causes abnormal pigmentation in Paralichthys olivaceus [37,38]. Additionally, blue light significantly enhances skin lightness in Pagrus pagrus [39], which is consistent with our present findings. The vibrant red, orange, and yellow colors on the surface of aquatic animals typically originate from the deposition of carotenoids, which mainly include α-carotene, *β*-carotene, astaxanthin, and lutein [40,41]. Our study further discovered that short-term blue-light exposure facilitated the accumulation of carotenoids and lutein in all-red-*koi* skin. Previous work has illustrated that lutein promotes yellow pigmentation and higher L* values through intrinsic pigment coloration [42,43]. Nevertheless, despite the improved skin lightness under light-spectrum treatments, long-term spectral exposure induced histological damage including tissue loosening and vacuolization in all-red-*koi* skin, which were most severe under red-light conditions. Furthermore, the ventral skin was more vulnerable to injury than dorsal skin. These findings suggest that favorable ornamental color performance does not guarantee healthy skin tissue in ornamental fish.

Transcriptomic analysis further elucidated the molecular mechanisms underlying the response of all-red *koi* to blue light. Differentially expressed genes were significantly enriched in environmental information processing pathways, indicating that light signals are primarily transduced via intracellular signal cascades rather than merely through metabolic alterations. This study identified three interacting core signaling pathways: the calcium signaling pathway, the MAPK signaling pathway, and the TGF-*β* signaling pathway. Our results suggest that these pathways are associated with the molecular response of all-red koi to blue light. Previous studies have shown that the calcium signaling pathway participates in the regulation of mammalian melanogenesis via multiple compartments, including the plasma membrane, endoplasmic reticulum, mitochondria, lysosomes/melanosomes and intercellular spaces [44]. The MAPK pathway modulates diverse cellular functions such as gene expression, proliferation and differentiation, and mediates cellular responses to various stimuli including ROS, pro-inflammatory cytokines and ultraviolet radiation [45,46]. As a vital anti-inflammatory factor, TGF-*β* forms the first line of defense against pathogen invasion in fish [47]. It is mainly responsible for immune homeostasis, tissue repair, cell differentiation and fibrosis. Moderate activation of TGF-*β* also plays an essential role in enhancing organismal immunity [48,49].

In this study, transcriptomic enrichment and PPI analyses suggest that the calcium signaling pathway may serve as a potential upstream signaling candidate linked to photic responses in fish pigment cells, potentially modulating downstream MAPK cascades. This correlative pathway pattern provides a hypothetical mechanism explaining short-term blue-light-associated pigment cell differentiation and carotenoid uptake. Furthermore, our gene expression data indicate that prolonged blue light exposure is transcriptionally correlated with altered TGF-*β* pathway activity, which may potentially regulate fibroblast function and extracellular matrix remodeling. The observed transcriptional changes associated with TGF-*β* signaling imply putative pathway overactivation, which coincided with disordered dermal collagen arrangement and connective tissue vacuolization, potentially contributing to the detected skin tissue phenotypic alterations. In addition, the downregulation of pigment synthesis-related genes (e.g., *pax*7, *cd*36, *scarb*, etc.) provides a transcriptome-based hypothetical explanation for the gradual attenuation of blue-light-induced pigment accumulation benefits.

## 5. Conclusions

Blue light appears to be an effective light source for short-term color enhancement. Moderate blue light exposure (30–60 days) significantly increases skin lightness (L* value), promotes the deposition of lutein and *β*-carotene, and upregulates the expression of multiple body color-related genes. In contrast, long-term exposure to red and white light was associated with potential drawbacks, such as inhibited growth and loosening of dermal tissue. Yellow light appears relatively beneficial to fish health but provides limited color enhancement. Based on these findings, we hypothesize that a cyclic lighting regimen of “short-term blue light for color enhancement followed by natural/yellow light for recovery” could serve as a potential management strategy. While this cyclic approach remains to be validated in future studies, our results provide a theoretical basis for optimizing lighting strategies in ornamental fish aquaculture.

## Figures and Tables

**Figure 1:**
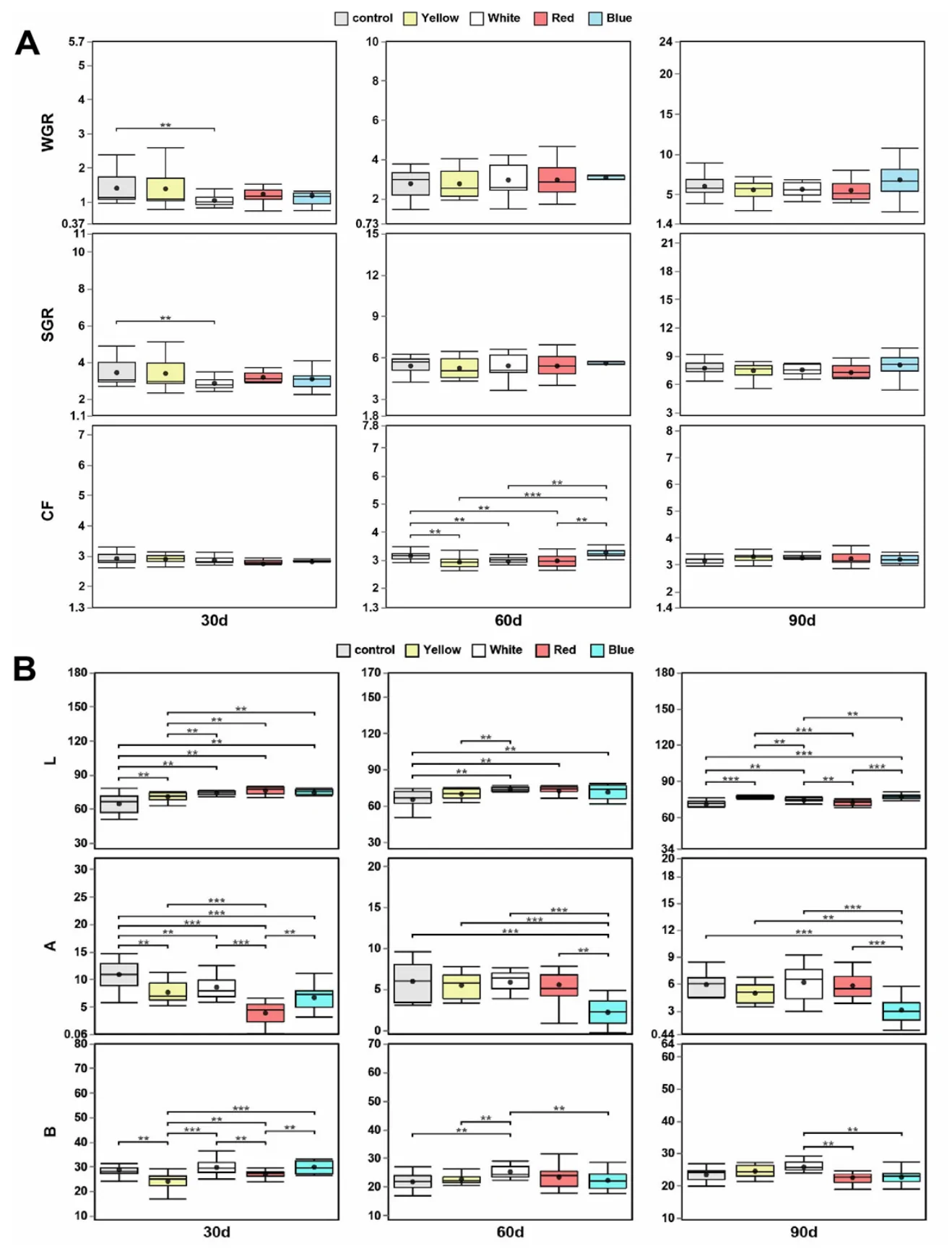
Effects of different light spectra on growth performance and skin color parameters of all-red *koi*. (**A**) Weight gain rate (WGR), specific growth rate (SGR) and condition factor (CF) of *koi* reared under different light spectra for 30, 60 and 90 days. (**B**) Color parameters L* (lightness), a* (redness), b* (yellowness) in dorsal skin. Control: NL; Yellow: YL; White: WL; Red: RL; Blue: BL. Data are shown as mean ± SD (n = number of replicate tanks; individual fish values within each tank were averaged). Significant differences between groups are indicated by asterisks (** *p* < 0.01, *** *p* < 0.001).

**Figure 2:**
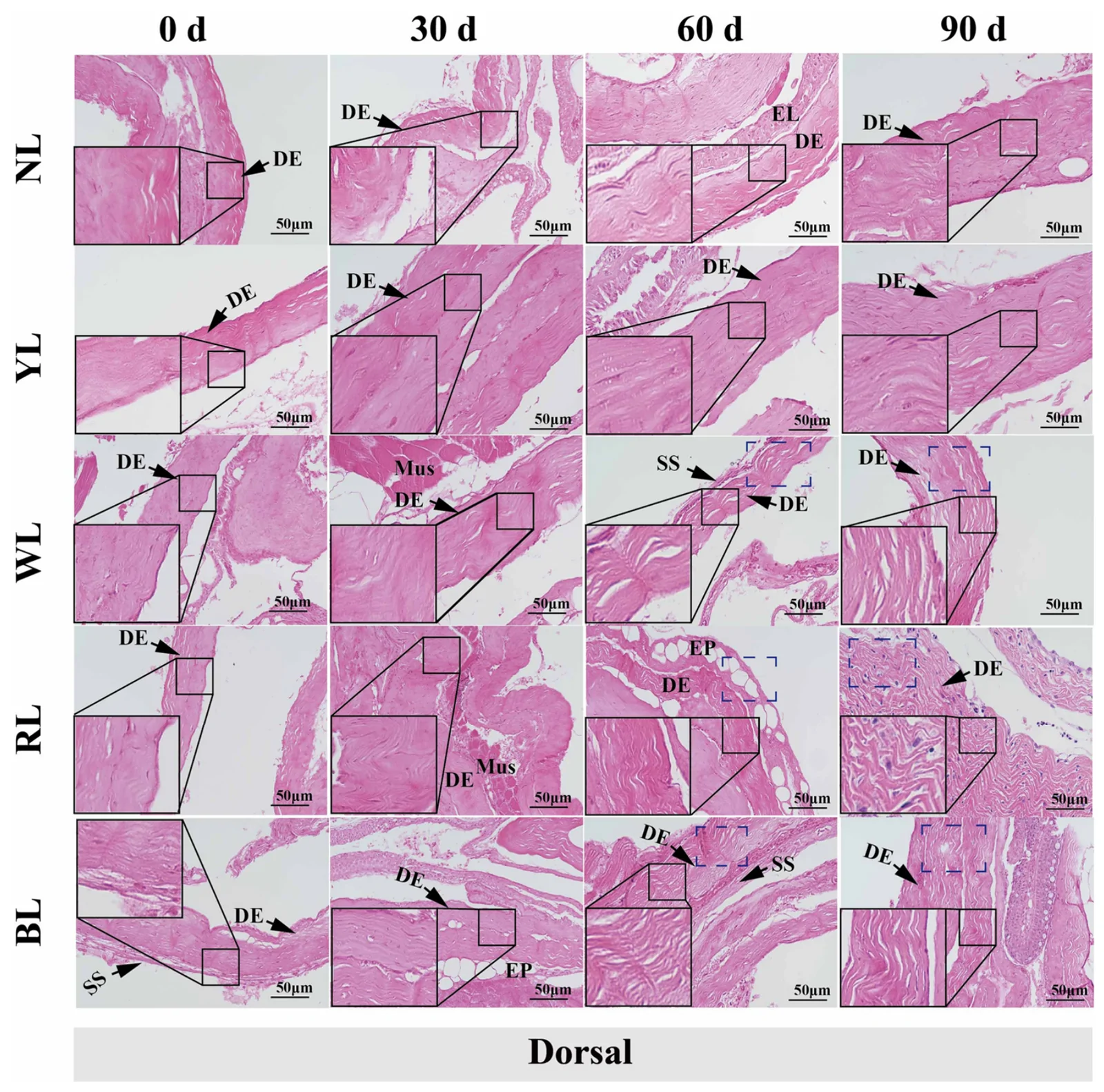
Histological observation of dorsal skin of all-red *koi* under different light spectra at 30, 60 and 90 days of culture (Hematoxylin-eosin staining). EP: Epidermis; DE: dermis; Mus: muscle; SS: stratum spongiosum. The enlarged insets highlight light-induced histological alterations in the dermal layers. Dotted rectangular boxes mark representative regions featuring obvious vacuolation expansion and tissue structural damage. Columns indicate sampling time points (30 days, 60 days, 90 days). Scale bar = 50 μm.

**Figure 3:**
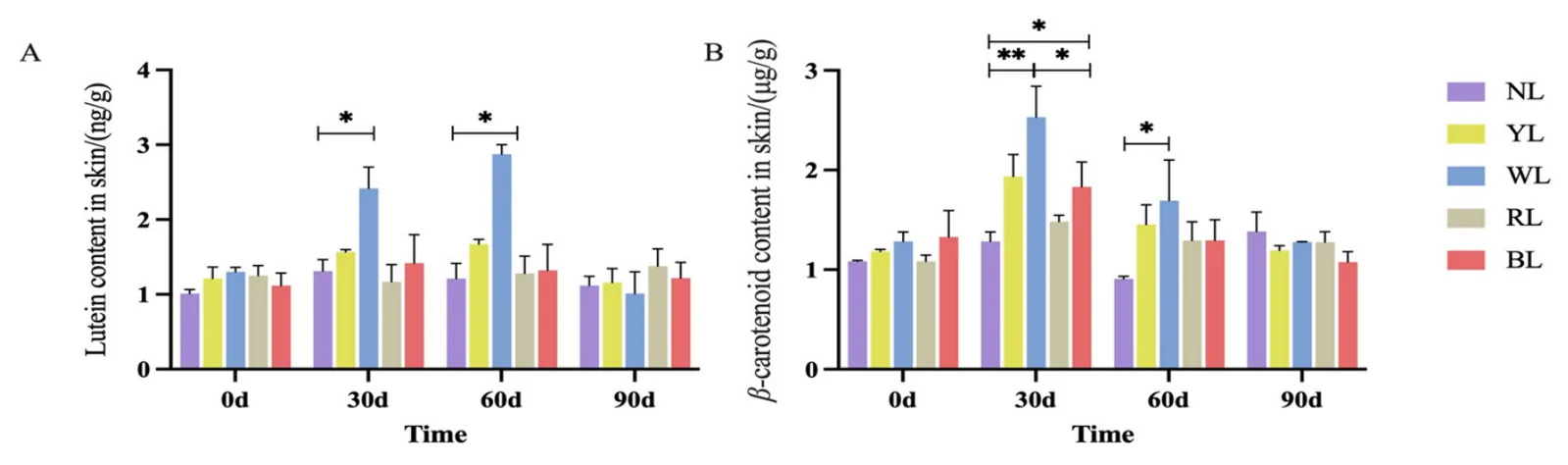
Changes in lutein and *β*-carotene contents in the skin of all-red *koi* under different light spectra. (**A**) Lutein content; (**B**) *β*-carotene content. Data are shown as mean ± SD (n = 3 biological replicates). Asterisks indicate significant differences between groups (* *p* < 0.05, ** *p* < 0.01).

**Figure 4:**
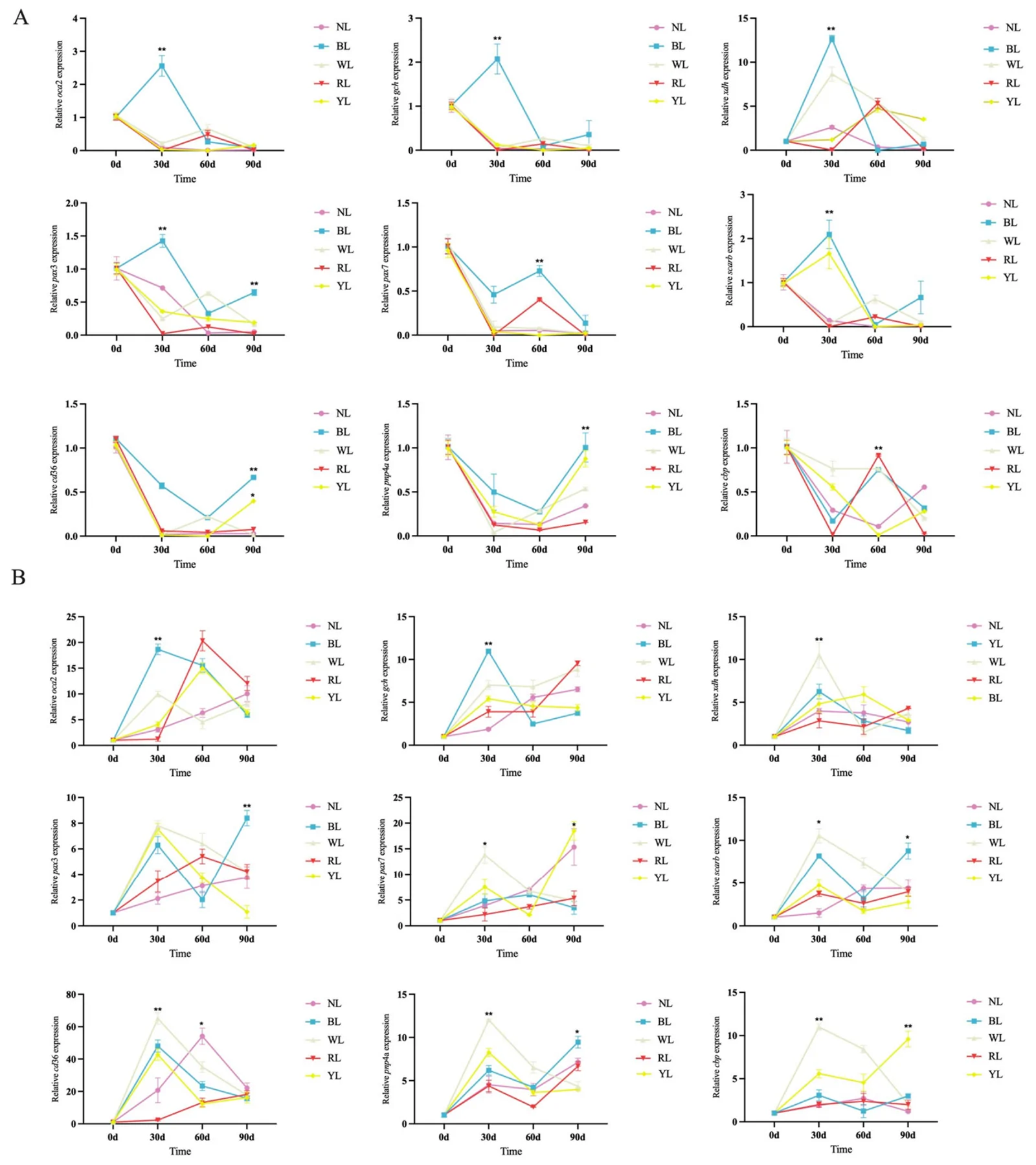
Relative mRNA expression levels of pigment-related genes in dorsal (**A**) and ventral (**B**) skin of all-red *koi* exposed to different light-spectrum treatments at 0, 30, 60 and 90 days. Data are shown as mean ± SD (n = 3 biological replicates). * *p* < 0.05, ** *p* < 0.01.

**Figure 5:**
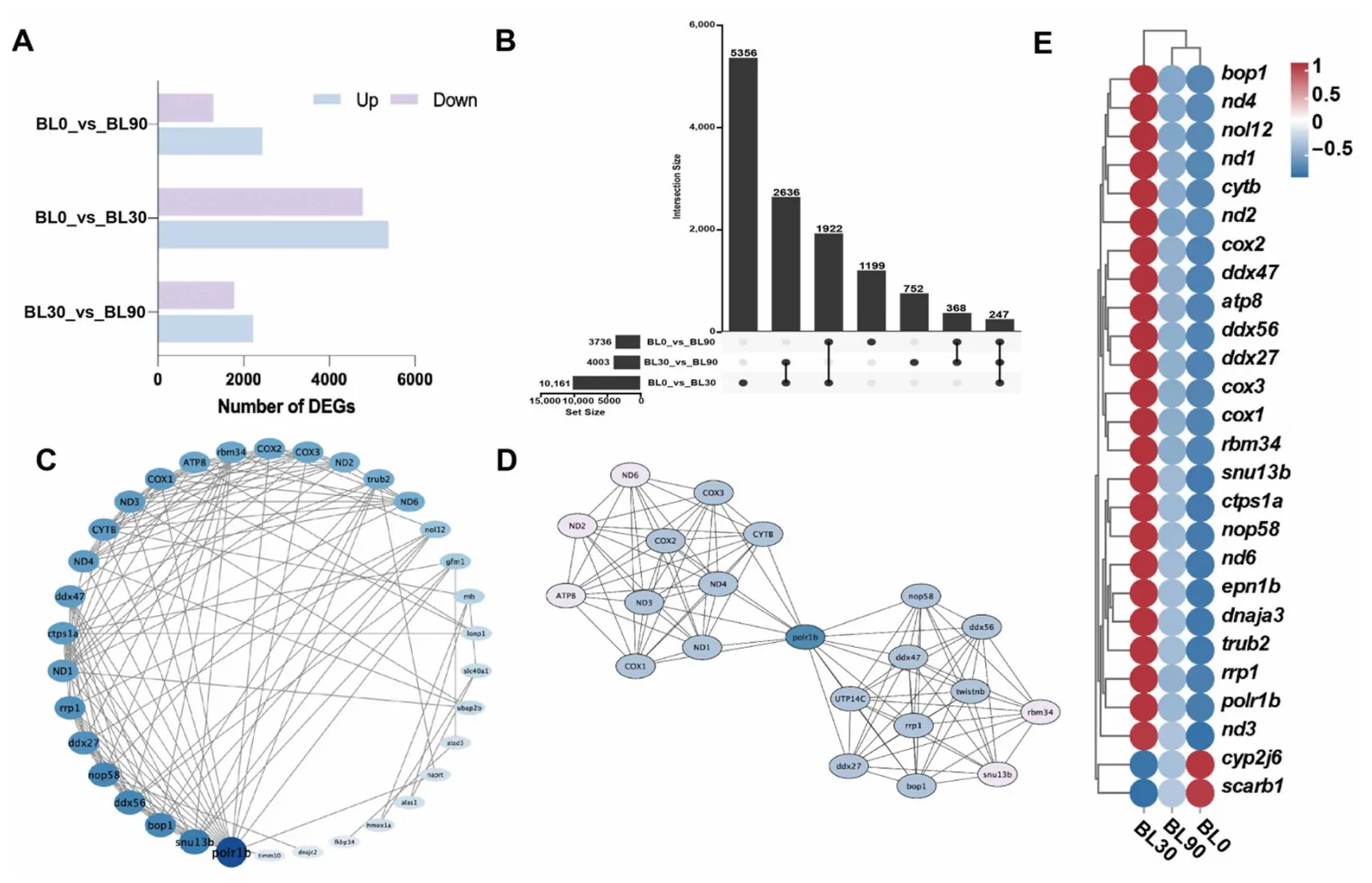
Transcriptome analysis of DEGs in *koi*. (**A**) The number of DEGs obtained from the RNA-seq analysis. (**B**) Upset diagram of the DEGs in various comparisons. (**C**) Regulatory network diagram of the mcode method. (**D**) Regulatory network diagram of the Cytohubba method. The color or size of nodes indicates connectivity, blue, pale blue and pale gray indicate high, medium, and low connectivity, respectively, and darker nodes indicate stronger connectivity. (**E**) Expression levels of 26 shared DEGs.

**Figure 6:**
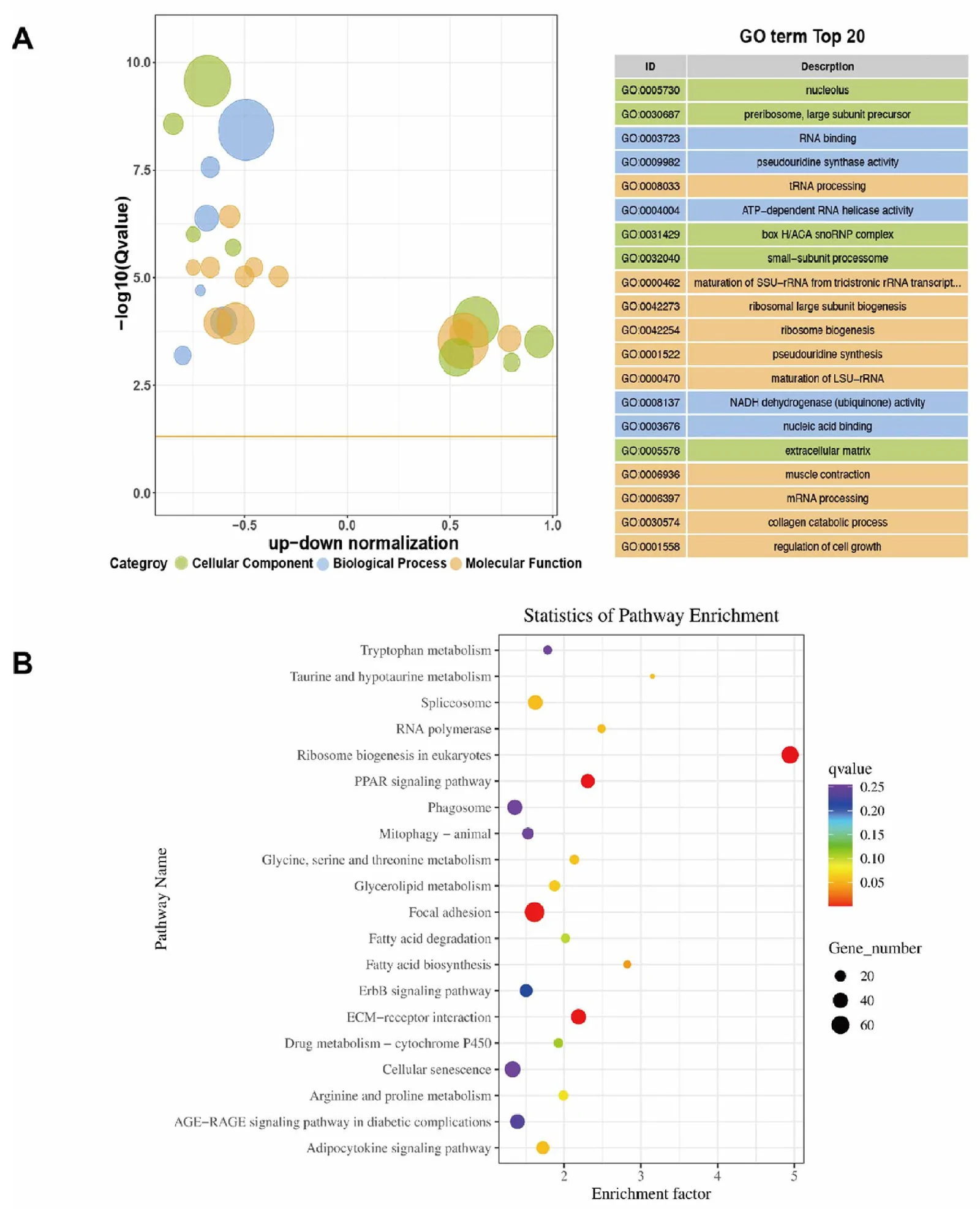
Functional enrichment information for DEGs. GO enrichment pie chart (**A**) and KEGG enrichment (**B**) difference bubble plot of BL30_vs_BL90 groups. The vertical coordinate is −log10(Q/*p* value), and the horizontal coordinate is the up–down normalization value (the ratio of the difference between the number of differentially upregulated genes and the number of differentially downregulated genes to the total number of differentially regulated genes); the size of the bubbles indicates the number of target genes enriched to the current term (pathway); the yellow line represents the threshold of Q/*p* value = 0.05. The right side is the list of top 20 term with Q value, different colors represent different Ontology.

**Figure 7:**
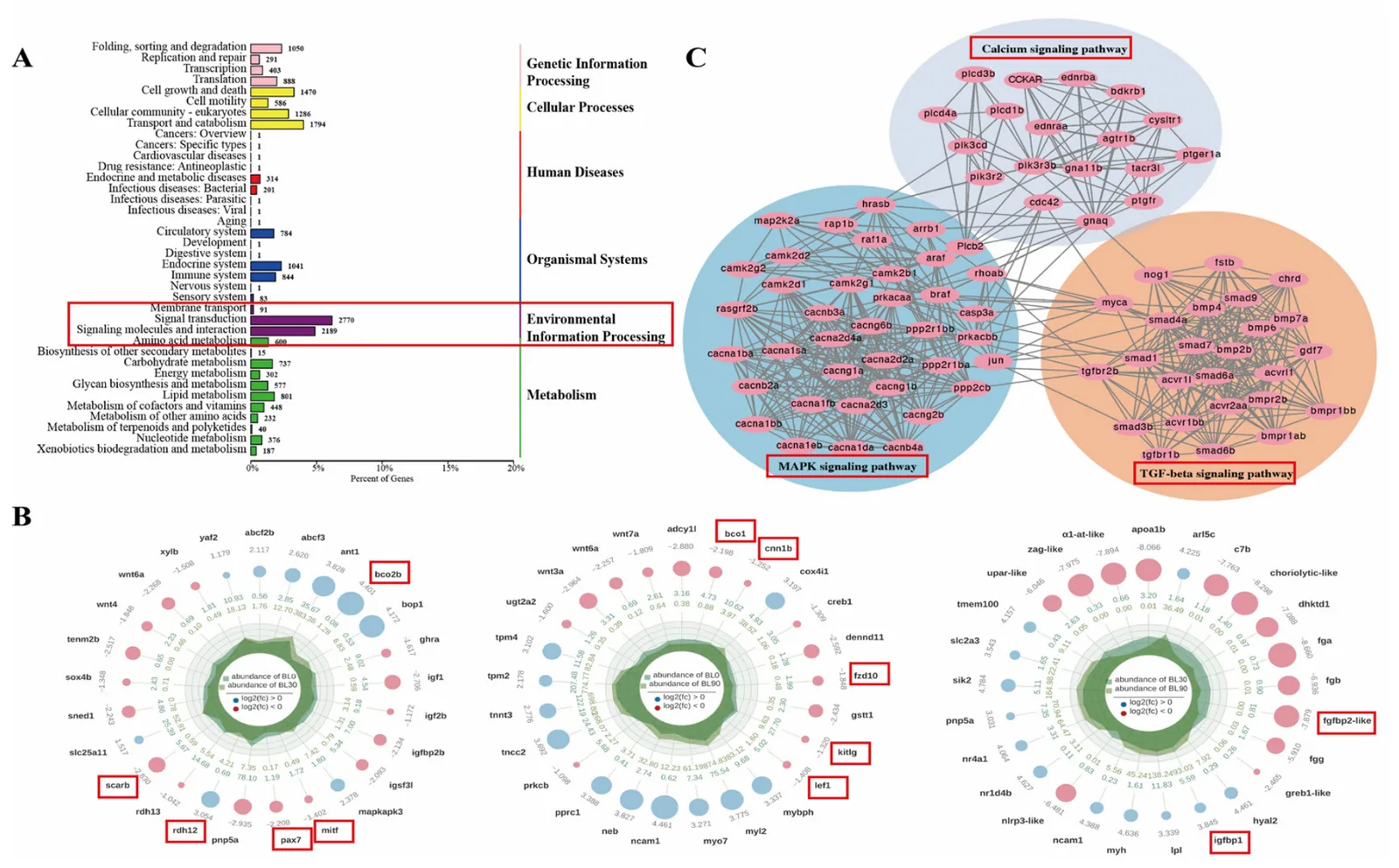
Protein–Protein Interaction (PPI) network analysis. (**A**) Top genes among DEGs associated with skin color in BL0_vs_BL90, BL0_vs_BL30 and BL30_vs_BL90 comparisons. (**B**) Top genes among DEGs associated with skin color in BL0_vs_BL90, BL0_vs_BL30 and BL30_vs_BL90 comparisons. (**C**) PPI network diagram of some DEGs. Boxed areas indicate key regions emphasized in the main text.

## Data Availability

The original contributions presented in the study are publicly available. All raw transcriptomic sequencing data have been deposited in the NCBI Sequence Read Archive (SRA) with the accession numbers PRJNA1509481.

## Supplementary Materials

The following supporting information can be downloaded at: https://www.mdpi.com/article/10.3390/ani16193022/s1, Figure S1: Effects of different light spectra on skin color parameters of all-red *koi*. Color parameters L* (lightness), a* (redness), b* (yellowness) in ventral skin (A) and fin section (B), respectively. Control: NL; Yellow: YL; White: WL; Red: RL; Blue: BL. Significant differences between groups are indicated by asterisks (* *p* < 0.05, ** *p* < 0.01, *** *p* < 0.001); Figure S2: Histological observation of ventral skin of all-red *koi* under different light spectra at 30, 60 and 90 days of culture (Hematoxylin-eosin staining). EP: Epidermis; DE: dermis; Mus: muscle; SS: stratum spongiosum. Dotted rectangular boxes mark representative regions featuring obvious vacuolation expansion and tissue structural damage. Columns indicate sampling time points (30 d, 60 d, 90 d). Scale bar = 50 μm; Figure S3: (A) Expression level and correlation analysis of samples. The lower-left panel shows pairwise scatter plots of gene expression levels between samples (x- and y-axes: log_2_(FPKM)). The upper-right panel displays the correlation heatmap among samples, where color intensity and numerical values indicate the degree of correlation. The middle panel presents expression density curves for each sample, with the *x*-axis representing log_10_(FPKM) and the *y*-axis representing the density value. (B) Principal-component analysis (PCA) of samples. PC1 denotes the first principal component, with the percentage in parentheses indicating its contribution rate to the variance among samples; Figure S4: Statistical results of differentially expressed genes annotation to various databases; Figure S5: (A) GO enrichment pie chart and KEGG enrichment difference bubble plot in the comparison BL0_vs_BL90 groups. (B) GO enrichment pie chart and KEGG enrichment difference bubble plot in the comparison BL0_vs_BL30 groups. Figure S6: Expression comparison of the selected genes by RNA-Seq and qPCR; Table S1: Primers used in the experiment; Table S2: RNA-seq data quality control result statistics.
