# Supplementary material for: Effects of Different Light Spectra on Growth Performance, Body Coloration, and Pigmentation-Related Gene Expression in Koi Carp (Cyprinus carpio var. koi)
Source: Animals (Basel). 2026 Sep 25;16(19):3022. doi: 10.3390/ani16193022 (PMC13663390; doi:10.3390/ani16193022)
Supplement: Supplementary file 1 [file animals-16-03022-s001.zip › Figure S1.pdf]

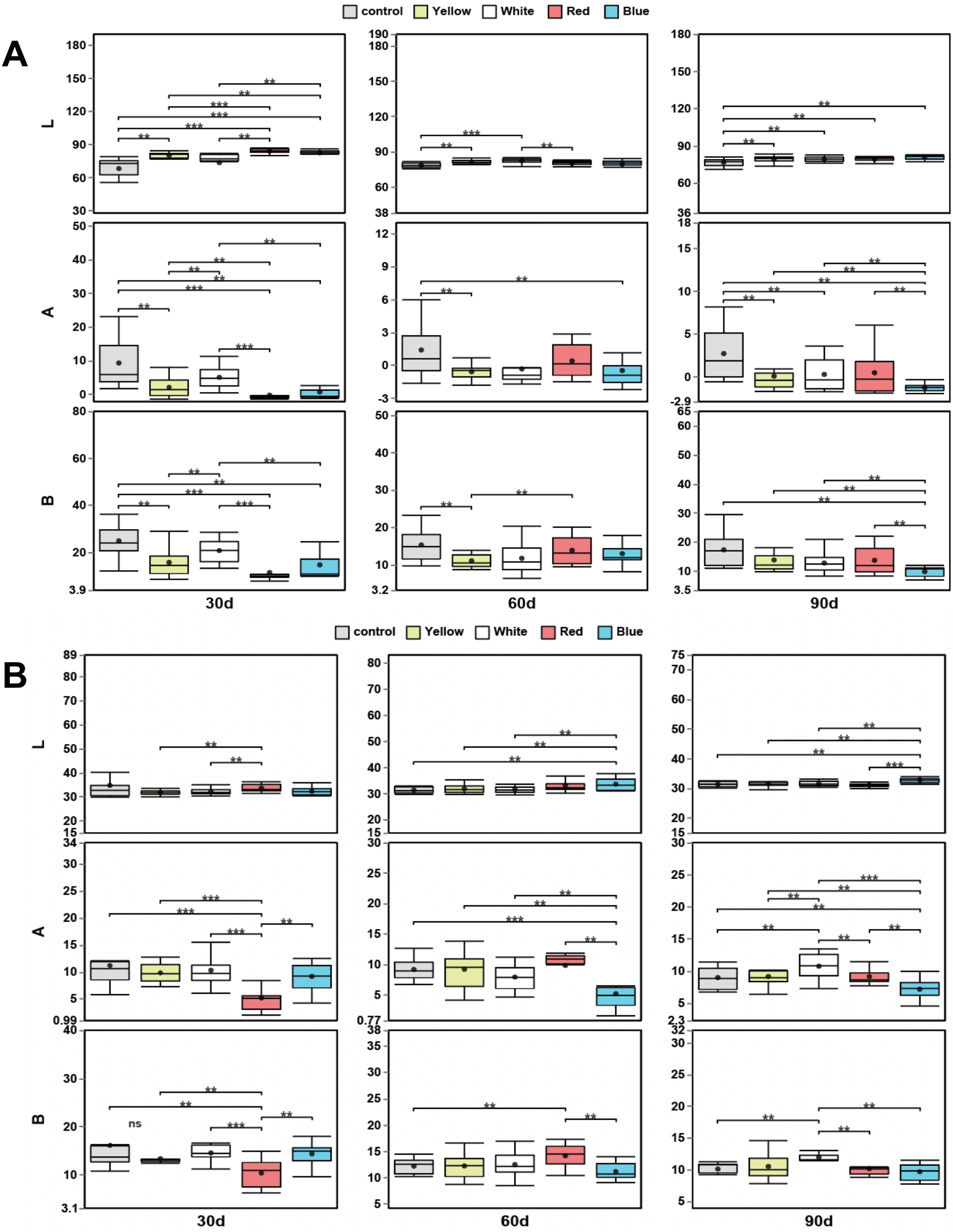
A
B
control
Yellow
White
Red
Blue
L
A
B
30d
60d
90d
ns
