# Supplementary material for: Effects of Different Light Spectra on Growth Performance, Body Coloration, and Pigmentation-Related Gene Expression in Koi Carp (Cyprinus carpio var. koi)
Source: Animals (Basel). 2026 Sep 25;16(19):3022. doi: 10.3390/ani16193022 (PMC13663390; doi:10.3390/ani16193022)
Supplement: Supplementary file 1 [file animals-16-03022-s001.zip › Figure S2.pdf]

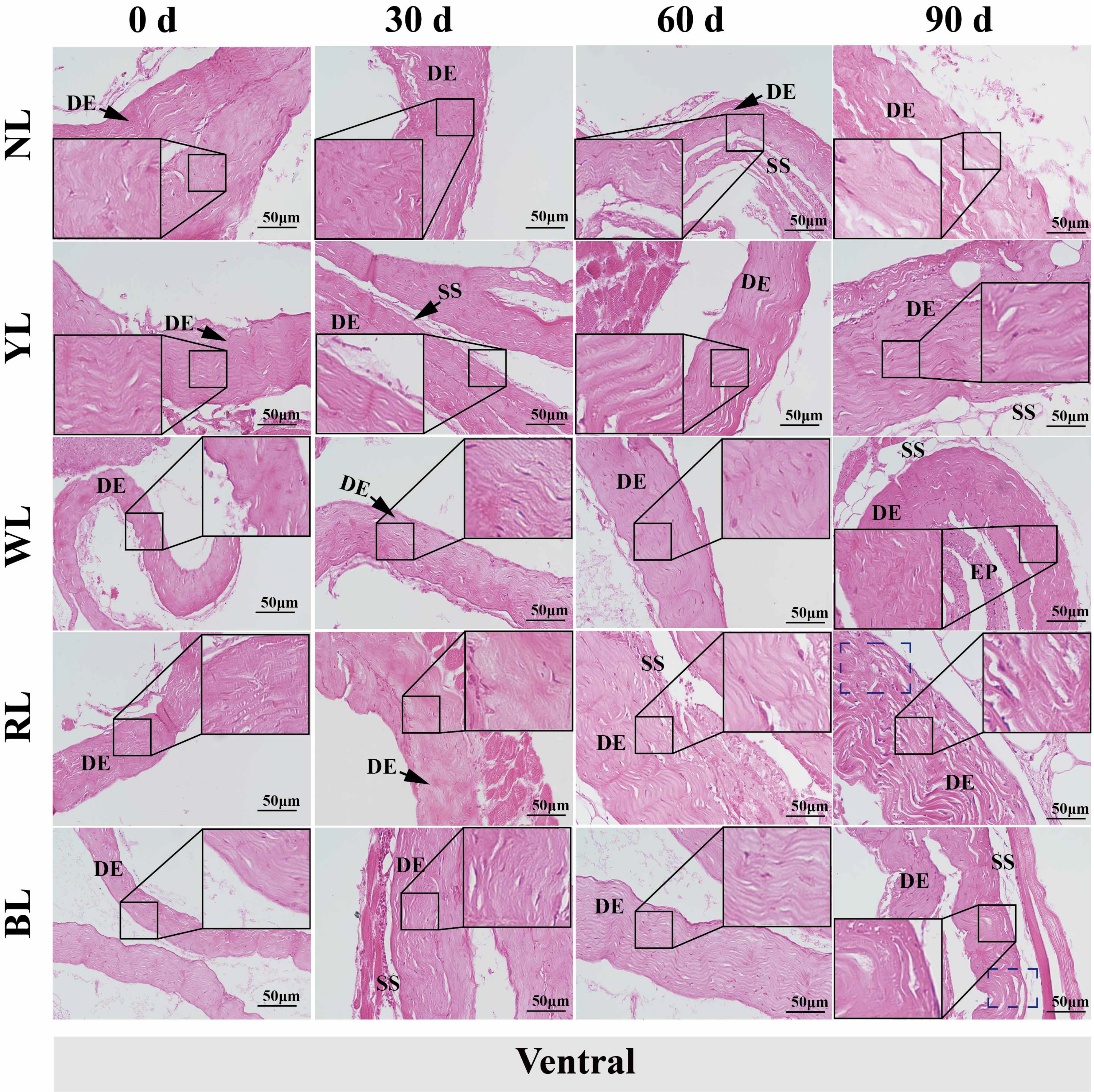

0 d
30 d
60 d
90 d
NL
YL
WL
RL
BL
DE
SS
EP
50μm
Ventral
