# Supplementary material for: Effects of Different Light Spectra on Growth Performance, Body Coloration, and Pigmentation-Related Gene Expression in Koi Carp (Cyprinus carpio var. koi)
Source: Animals (Basel). 2026 Sep 25;16(19):3022. doi: 10.3390/ani16193022 (PMC13663390; doi:10.3390/ani16193022)
Supplement: Supplementary file 1 [file animals-16-03022-s001.zip › Figure S3.pdf]

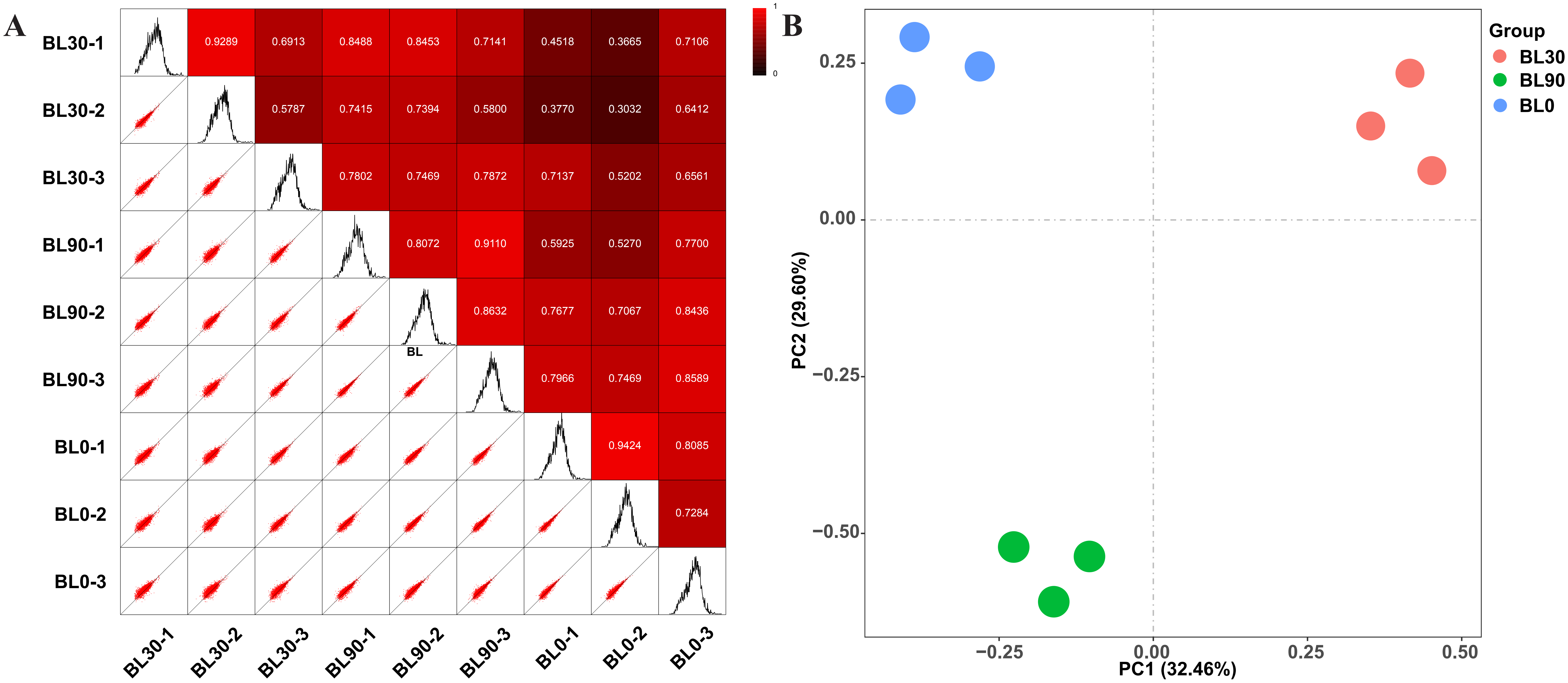

A
BL30-1
BL30-2
BL30-3
BL90-1
BL90-2
BL90-3
BL0-1
BL0-2
BL0-3
0.9289
0.6913
0.8488
0.8453
0.7141
0.4518
0.3665
0.7106
0.5787
0.7415
0.7394
0.5800
0.3770
0.3032
0.6412
0.7802
0.7469
0.7872
0.7137
0.5202
0.6561
0.8072
0.9110
0.5925
0.5270
0.7700
0.8632
0.7677
0.7067
0.8436
BL
0.7966
0.7469
0.8589
0.9424
0.8085
0.7284
1
0
B
Group
BL30
BL90
BL0
0.25
0.00
−0.25
−0.50
PC2 (29.60%)
−0.25
0.00
0.25
0.50
PC1 (32.46%)
