# Supplementary material for: Effects of Different Light Spectra on Growth Performance, Body Coloration, and Pigmentation-Related Gene Expression in Koi Carp (Cyprinus carpio var. koi)
Source: Animals (Basel). 2026 Sep 25;16(19):3022. doi: 10.3390/ani16193022 (PMC13663390; doi:10.3390/ani16193022)
Supplement: Supplementary file 1 [file animals-16-03022-s001.zip › Figure S4.pdf]

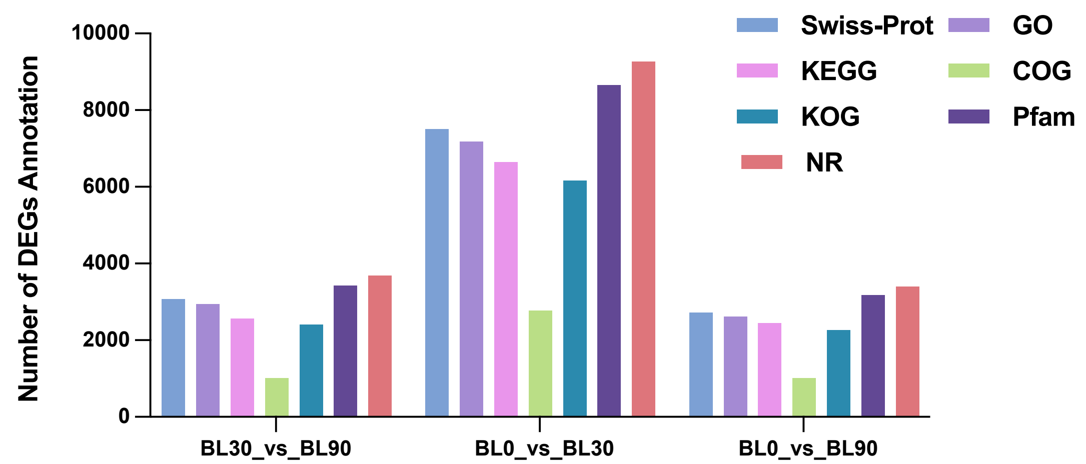
Number of DEGs Annotation
10000
8000
6000
4000
2000
0
BL30_vs_BL90
BL0_vs_BL30
BL0_vs_BL90
Swiss-Prot
GO
KEGG
COG
KOG
Pfam
NR
