# Supplementary material for: Effects of Different Light Spectra on Growth Performance, Body Coloration, and Pigmentation-Related Gene Expression in Koi Carp (Cyprinus carpio var. koi)
Source: Animals (Basel). 2026 Sep 25;16(19):3022. doi: 10.3390/ani16193022 (PMC13663390; doi:10.3390/ani16193022)

A

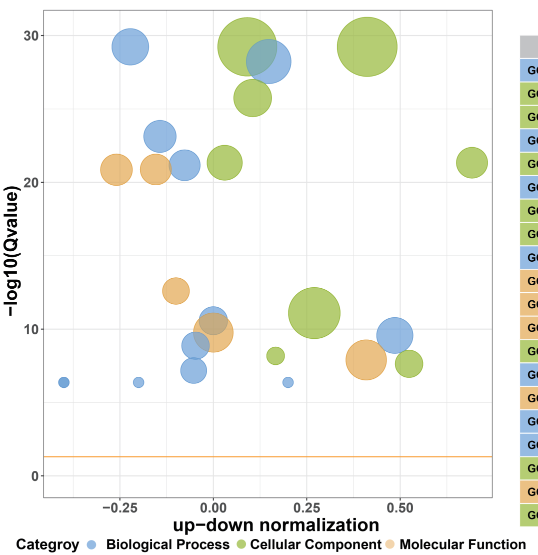

GO term Top 20

ID	Descrption
GO:0007015	actin filament organization
GO:0005743	mitochondrial inner membrane
GO:0005739	mitochondrion
GO:0045214	sarcomere organization
GO:0031430	M band
GO:0071688	striated muscle myosin thick filament assemb
GO:0005859	muscle myosin complex
GO:0005861	troponin complex
GO:0006941	striated muscle contraction
GO:0097493	structural molecule activity conferring elastic
GO:0051371	muscle alpha−actinin binding
GO:0004129	cytochrome−c oxidase activity
GO:0030018	Z disc
GO:0006099	tricarboxylic acid cycle
GO:0051015	actin filament binding
GO:0006936	muscle contraction
GO:0006096	glycolytic process
GO:0070469	respiratory chain
GO:0008307	structural constituent of muscle
GO:0031672	A band

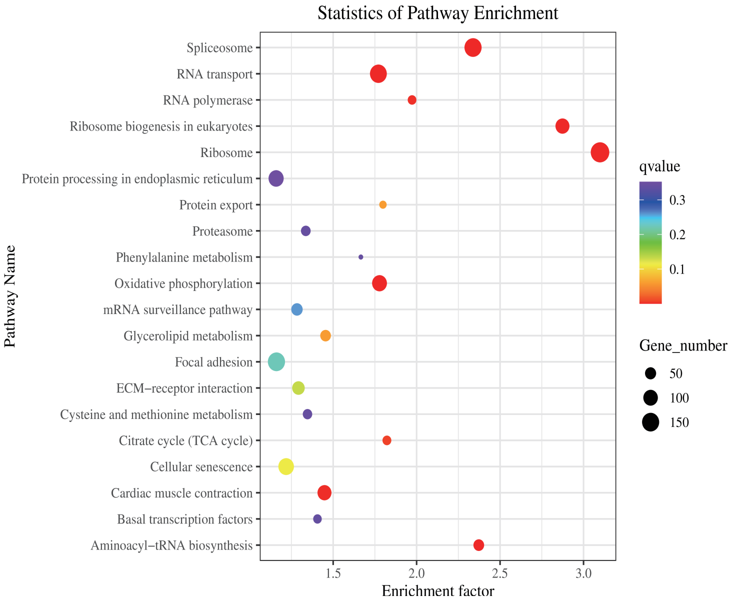

B

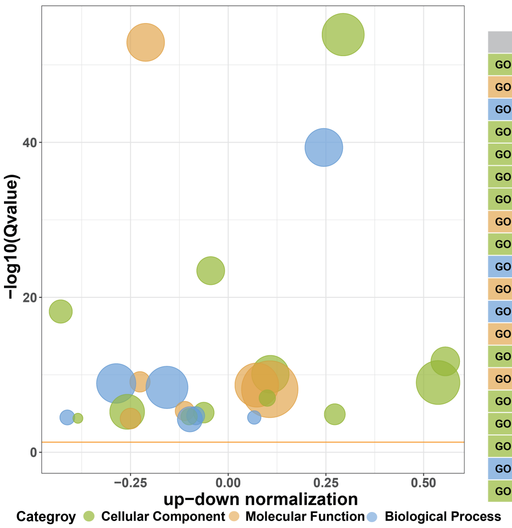

GO term Top 20

ID	Descrption
GO:0005730	nucleolus
GO:0003735	structural constituent of ribosome
GO:0006412	translation
GO:0005840	ribosome
GO:0022625	cytosolic large ribosomal subunit
GO:0005681	spliceosomal complex
GO:0005743	mitochondrial inner membrane
GO:0019843	rRNA binding
GO:0005739	mitochondrion
GO:0008380	RNA splicing
GO:0000166	nucleotide binding
GO:0006397	mRNA processing
GO:0003723	RNA binding
GO:0030687	preribosome, large subunit precursor
GO:0000049	tRNA binding
GO:0016607	nuclear speck
GO:0033017	sarcoplasmic reticulum membrane
GO:0005685	U1 snRNP
GO:0000381	regulation of alternative mRNA splicing, via spliceosor
GO:0005762	mitochondrial large ribosomal subunit

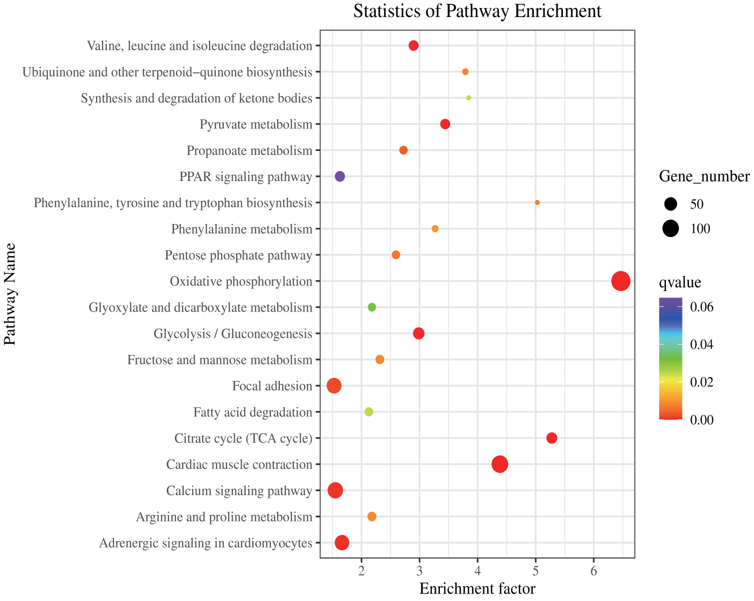

Supplement: Supplementary file 1 [file animals-16-03022-s001.zip › Figure S5.pdf]
