# Supplementary material for: Effects of Different Light Spectra on Growth Performance, Body Coloration, and Pigmentation-Related Gene Expression in Koi Carp (Cyprinus carpio var. koi)
Source: Animals (Basel). 2026 Sep 25;16(19):3022. doi: 10.3390/ani16193022 (PMC13663390; doi:10.3390/ani16193022)

Comparison group	abbreviation	KEGG annotation	Gene annotation	up/down
BL0_vs_BL90	*mb*	Energy production and conversion	myoglobin	up
	tnnt 3b	Cytoskeleton	troponin T, fast skeletal muscle isoforms-like isoform X5	up
	eno 3	Carbohydrate transport and metabolism	beta-enolase	up
	hspb 7	Posttranslational modification, protein turnover, chaperones	heat shock protein beta-7-like	up
	tpm 2	Cytoskeleton	tropomyosin alpha-3 chain isoform X7	up
BL30_vs_BL90	*zbed* 4	Replication, recombination and repair	zinc finger BED domain-containing protein 4-like	down
	lpl	PPAR signaling pathway	lipolipase 1b	up
	pnp 5a	Nucleotide transport and metabolism	purine nucleoside phosphorylase-like	up
	hsp 90*a* 1	Posttranslational modification, protein turnover, chaperones	heat shock protein HSP 90-alpha 1	up
	hmox 1*a*	Inorganic ion transport and metabolism	heme oxygenase-like	up
	bop 1	Translation, ribosomal structure and biogenesis	ribosome biogenesis protein bop1	up
BL0_vs_BL30	*dnajc* 2	Posttranslational modification, protein turnover, chaperones	dnaJ homolog subfamily C member 2 isoform X1	up
	scarf 2	Signal transduction mechanisms	scavenger receptor class F member 2-like isoform X1	down
	lcmt 2	Posttranslational modification, protein turnover, chaperones	tRNA wybutosine-synthesizing protein 4	down
	ece 2*a*	Amino acid transport and metabolism	endothelin-converting enzyme 2-like isoform X3	down
	cabp 8	Signal transduction mechanisms	calcium-binding protein 8 isoform X2	down
	pygma	Carbohydrate transport and metabolism	glycogen phosphorylase, muscle form-like isoform X1	up
	mdn 1	General function prediction only	midasin-like	down

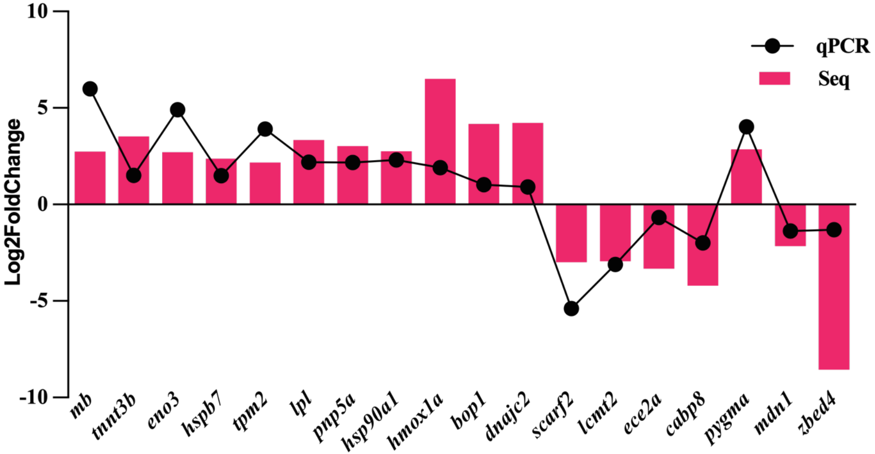

Supplement: Supplementary file 1 [file animals-16-03022-s001.zip › Figure S6.pdf]
